# Impact of Clinical Factors on Generic and Disease-Specific Quality of Life in COPD and Asthma-COPD Overlap with Exacerbations

**DOI:** 10.1155/2020/6164343

**Published:** 2020-06-25

**Authors:** Zsófia Lázár, Alpár Horváth, Gábor Tomisa, Lilla Tamási, Veronika Müller

**Affiliations:** ^1^Dept. Pulmonology, Semmelweis University, 25-29 Tömő Str. 1083 Budapest, Hungary; ^2^Chiesi Hungary Ltd., Dunavirág u. 2, 1138 Budapest, Hungary

## Abstract

**Purpose:**

The health-related quality of life (HRQL) in chronic obstructive pulmonary disease (COPD) is worsened by frequent exacerbations, and it can be affected by the concomitant presence of bronchial asthma (asthma-COPD overlap (ACO)). The impacts of clinical factors associated with HRQL have not been compared in patients with COPD and ACO experiencing exacerbations. *Patients and Methods*. Patients with COPD (*N* =705) and ACO (*N* =148) belonging to C and D groups according to GOLD 2017 were recruited in stable condition. Demographic and clinical data were collected, spirometry was performed, and patients rated the intensity of respiratory symptoms during the previous week. The COPD Assessment Test (CAT) and the EQ-5D 3 level version (dimensions and visual analogue scale (VAS)) were used to assess disease-specific and generic HRQL, respectively. Fisher's exact test, *χ*^2^ test, ANOVA, and Pearson correlation were used for analysis (mean ± SD). Multiple linear regression was applied to identify variables related to CAT and EQ-5D VAS scores.

**Results:**

The CAT and EQ-5D VAS scores showed similarly low HRQL in COPD and ACO (20.7 ± 6.7 vs. 21.1 ± 6.3 (*p* = 0.52) and 56.2 ± 17.8 vs. 53.7 ± 18.2 (*p* = 0.11)). There was a weak correlation between CAT and EQ-5D VAS scores (COPD: *r* = −0.345, *p* < 0.001; ACO: *r* = −0.245, *p* = 0.003). More patients with COPD had problems related to anxiety/depression in EQ-5D (63.7% vs. 55.4%, *p* = 0.06). Pack-years exerted a negative effect on HRQL measures both in ACO and COPD. Low HRQL in COPD was associated with female gender, dyspnea, cough, gastroesophageal reflux disease, and arrhythmia, while in ACO, it was related to arrhythmia, hypertension, and cough, but less to dyspnea.

**Conclusions:**

Patients with COPD and ACO experiencing exacerbations have low quality of life, which is influenced by smoking history, symptoms, and comorbidities. These findings have important implications for the development of therapeutic strategies to improve the health status of patients with these conditions.

## 1. Introduction

Chronic obstructive pulmonary disease (COPD) is characterized by irreversible and progressive airflow limitation, the presence of multiple comorbidities, and reduced quality of life [1]. Several factors have been described to be negatively related to the quality of life measures in COPD such as the lower level of education, an unskilled profession, female gender, and disease severity [2–4]. Comorbidities including peripheral arterial disease, osteoporosis, depression, diabetes, stroke, and myocardial infarction also exert an additional unfavorable effect on the health status [2, 5–7]. Exacerbations are important events in the course of COPD, and notably, patients with frequent exacerbations suffer from poorer quality of life [8, 9]. However, the influence of clinical factors on health status in frequent exacerbators has not been explored.

A subgroup of patients with COPD also show clinical features of bronchial asthma and can be termed to have asthma-COPD overlap (ACO) [10]. Patients with ACO are younger; they have a lower level of dyspnea and smoked less than patients with COPD alone [11]. Of note, the rate of exacerbation was shown to be unchanged [11, 12], but also increased [13, 14] in ACO. Compared to COPD, patients with ACO have more severe airway obstruction [15, 16], more severe wheezing [16], and a different set of comorbidities [17]. Reports on quality of life in ACO vs. COPD showed contradictory findings [12, 18], and health measures have not been studied in frequent exacerbators with overlap disease.

Thus, we aimed to investigate the health-related quality of life (HRQL) in patients with COPD and ACO who experience multiple disease relapses. We compared the clinical features and the disease-specific and a generic HRQL in patients with COPD and ACO belonging to groups C and D as classified according to the Global Initiative for Chronic Obstructive Lung Disease (GOLD) recommendation [1]. Furthermore, the influence of demographic and clinical variables, as well as comorbidities on the health status of patients, was also studied.

## 2. Material and Methods

### 2.1. Patients and Study Design

Patients were recruited by 71 respiratory specialists during regular control visits at outpatient pulmonology centers in Hungary between 01 November 2017 and 31 March 2018. Inclusion criteria were age > 18 years, COPD diagnosed >1 year ago by a respiratory specialist (as postbronchodilator FEV_1_/FVC < 0.70) [1], and exacerbation history equivalent to groups C or D according to GOLD document 2017 [1]. ACO was established when patients showed overlapping clinical characteristics of COPD and bronchial asthma [10] together with a concomitant diagnosis of bronchial asthma established at least one month prior to inclusion. Patients with ACO had a positive bronchial hyperreactivity or reversibility testing (>12% or 200 ml after the inhalation of 400 *μ*g salbutamol) in the past. Exclusion criteria were the presence of severe and untreated comorbidity, inability to fill out the HRQL questionnaires, current systemic steroid or antibiotic treatment, restrictive ventilatory failure, severe cardiac failure, current tuberculosis infection, and active cancer.

The study had a noninterventional longitudinal design. During the first visit, detailed clinical characteristics, information on drug side effects, spirometric variables, and data on symptoms and HRQL were collected, inhaler technique was checked and corrected when necessary. At later visits (after 1 month and 3 months), the inhaler technique was rechecked, education on inhaler use was repeated, and spirometric values, symptom scores, and HRQL variables were recorded. Data of patients with three completed visits were collected for statistical analysis (*N* = 944). During data clearance, 91 patients were excluded for not meeting the inclusion criteria for exacerbation number (*N* = 853). In the present work, data only from the first visit were analyzed in a cross-sectional manner.

The study was approved by the Hungarian Medical Research Council (46367-4/2017/EKU) and the National Institute of Pharmacy and Nutrition (46402-5/2017). Each patient signed a written informed consent. All work was conducted in accordance with the Declaration of Helsinki (1964).

### 2.2. Data Collection

Demographic and basic clinical data were recorded (age, gender, smoking history, number of moderate, and severe past exacerbations according to categories). Former smoking was defined as smoking stopped at least 6 months earlier. Educational level was noted as completed higher education (higher), completed secondary education (middle), and completed or uncompleted primary education (low). Height, weight, and prebronchodilator spirometry (FVC, FEV_1_) were measured [19]. Body mass index (BMI) was calculated, and patients were categorized into four groups [20]: underweight (<20 kg/m^2^), normal weight (20 to 24.9 kg/m^2^), overweight (25 to 29.9 kg/m^2^), and obese (≥30 kg/m^2^). Current inhaled therapy regimens (classes of drugs) and the use of short-acting bronchodilators in the past week (≤1/week, 2-3/week, >3/week) were recorded.

Self-reported comorbidities were noted according to predefined categories (gastroesophageal reflux disease (GERD), ischemic heart disease, hypertension, myocardial infarction, arrhythmia, heart failure, cerebrovascular event, osteoporosis, diabetes, glaucoma, and chronic bronchitis). Arrhythmia was defined as an existing chronic arrhythmia or a previous intermittent episode. A previous stroke or a past intermittent cerebrovascular episode was considered a cerebrovascular event. Chronic bronchitis was defined as at least 3 months of productive coughing in 2 or more consecutive years, and it was assessed only in patients with COPD.

Patients rated their level of dyspnea using the Modified British Medical Research Council (mMRC) Questionnaire [21], and they were asked to assess the intensity of symptoms (dyspnea at rest, dyspnea during daily activities, and cough) in the past week on a scale from 0 to 10, where 0 meant no symptoms and 10 referred to unbearable symptoms. Sputum production in the past week was also reported: no sputum (0), a small amount (1), a moderate amount (2), and a significant amount (3).

Quality of life was measured with the disease-specific COPD Assessment Test (CAT), which is a validated 8-item tool used for monitoring COPD [22]. Symptoms were self-assessed by patients on a scale from 0 to 5; the maximal overall score is 40 with higher scores referring to higher symptom burden.

The EQ-5D-3L (3 level version) and the EQ-5D-VAS (visual analogue scale) were applied to evaluate the generic health status [23]. The EQ-5D-3L comprises of 5 dimensions: mobility, self-care, usual activities, pain/discomfort, and anxiety/depression. In each dimension, the disability can be rated by the patients on 3 levels: no problems (1), some problems (2), and extreme problems (3). Due to the lack of available value sets representative for the Hungarian population, a utility score could not be calculated; therefore, the dimensions were individually analyzed. Patients graded their today health using the VAS in the form of a 20 cm vertical line ranging from 0 to 100, where 100 represents the best imaginable and 0 the worst imaginable health status.

### 2.3. Data Analysis

Categorical variables were analyzed with the Fisher exact and *χ*^2^ tests (combined with post hoc analysis). Continuous variables showed Gaussian distribution and were analyzed using a *t*-test, ANOVA, and Pearson correlation and presented through their means and standard deviations (SD). Multiple linear regression was applied to identify clinical variables related to CAT and EQ-5D VAS. The SPSS 19.0. software (IBM, Armonk, NY, US) was applied for statistical analysis; *p* values < 0.05 were considered significant.

## 3. Results

### 3.1. Demographic Variables in Patients with COPD and ACO

Among the 853 patients included in the study, 18% and 82% had ACO and COPD alone, respectively (Table 1). Most patients belonged to GOLD group D (COPD patients C/D: 5.0/95.0%, ACO patients: group C/D: 3.4%/96.6%; *p* = 0.41). There was a trend for more male patients with COPD. The BMI was higher in patients with ACO with an increased ratio of obese patients, while we observed a tendency for more underweight patients with COPD. As expected, there were more current smokers with COPD, and more patients were nonsmokers in the ACO group; however, the pack-years were similar in the two groups. FEV_1_ and FEV_1_/FVC were lower in COPD alone. Inhaled therapy regimens with ICS (inhaled corticosteroid) were more frequently used by patients with ACO, while long-acting muscarinic antagonist and long-acting beta_2_-agonist (LAMA+LABA) products were applied by more patients with COPD alone. We noted a trend for an increased number of hypertension cases and a higher overall burden of comorbidities in COPD. No difference was noted in age, educational level, and the number of COPD-related emergency room visits and hospitalizations between the two groups.

### 3.2. Quality of Life and Symptoms in Patients with COPD and ACO

**Figure 1:**
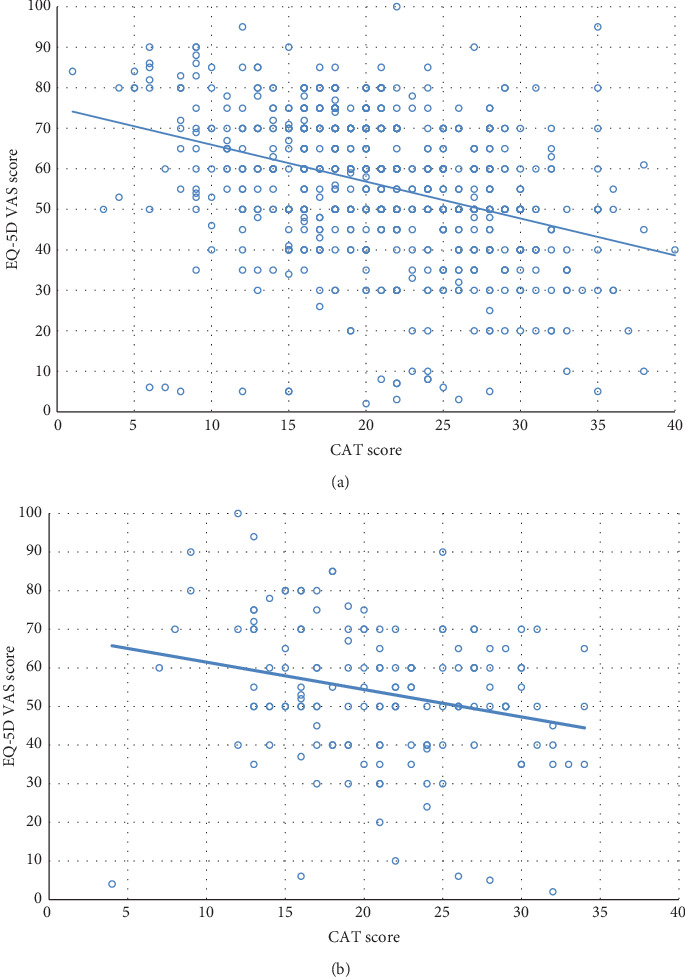
Correlation between the CAT and the EQ-5D VAS scores: (a) COPD, *r* = −0.345, *p* < 0.001; (b) ACO, *r* = −0.245, *p* = 0.003 (Pearson correlation).

There was a significant but weak correlation between CAT and EQ-5D VAS scores in patients with COPD (*r* = −0.345, *p* < 0.001; Figure 1(a)) and ACO (*r* = −0.245, *p* = 0.003; Figure 1(b)). The CAT score showed a similarly low disease-specific quality of life in both groups, which is line with the predominance of the GOLD D group patients in the populations (Table 2). The EQ-5D VAS tool demonstrated a mean decrease of 2.5 points in ACO compared to COPD; however, it did not reach statistical significance. A trend for more severe anxiety and depression was noted in patients with COPD, and the proportion of patients with any problems (some or extreme problems) in the anxiety/depression dimension was increased in COPD vs. ACO (63.7% vs. 55.4%, *p* = 0.06). We found no difference in the health status between the two groups analyzed according to other EQ-5D dimensions (Table 2).

The intensity of respiratory symptoms and the use of short-acting bronchodilator in the past week were similar in the two groups (Table 2). Of note, approximately half of the patients needed to use a reliever medication more than three times in the previous week, highlighting the high symptom burden.

Interestingly, the CAT score was increased in patients with ACO on LAMA+LABA therapy compared to COPD patients on dual long-acting bronchodilators (Table 3). Data on mono-LAMA and mono-LABA use were not analyzed due to the low number of patients in the ACO group (*n* = 2 and *n* = 0, respectively).

### 3.3. Clinical Variables Associated with Health Status in Patients with COPD

In patients with COPD, a lower disease-specific quality of life, denoted by a higher CAT score, was associated with pack-years and the increased number of severe exacerbations in the past 12 months (Table 4). The intensity of dyspnea either at rest or during daily activities and the intensity of cough were related to higher CAT scores; however, the amount of sputum showed no association. Among the comorbidities, only GERD was associated with higher CAT scores.

As shown by the EQ-5D VAS score, a better generic HRQL was linked to a higher level of education, while it was negatively associated with female gender, pack-years, arrhythmia, and dyspnea at rest. There was a trend for worse health status in patients with the frequent use of reliever medications.

### 3.4. Clinical Variables Associated with Health Status in Patients with ACO

In patients with ACO, an increased CAT score was associated with the high intensity of cough in the past week (Table 4). In addition, the frequent use of reliever medications was related to higher CAT scores, and arrhythmia showed a trend for an increase in the CAT score. Unexpectedly, we found a tendency between lower CAT scores and a high number of previous severe COPD-related exacerbation and the presence of obesity (trend toward significance).

The EQ-5D VAS score was positively associated with a higher level of education. There was a negative relationship between the general health status and pack-years, arrhythmia, and the frequent use of short-acting bronchodilators. Moreover, we observed a trend for a negative association between the EQ-5D VAS score and hypertension and the intensity of dyspnea at rest.

## 4. Discussion

Exacerbations in COPD are associated with worse and deteriorating quality of life [8, 24]. A higher number of hospitalizations due to respiratory symptoms [13, 14] and a lower HRQL [18] were recorded in patients with ACO compared to subjects with asthma or COPD alone. The mechanism of ACO is currently incompletely understood, and more research is needed to identify its specific clinical and physiological characteristics [10]. In the present study, we focused on patients with COPD and ACO with a history of past exacerbations and found that both groups have similarly low HRQL, which is associated with partly shared clinical variables including the burden of respiratory symptoms and comorbidities.

The clinical characteristics of patients with ACO and COPD showed differences. We observed a higher number of women, increased BMI, and somewhat better FEV_1_/FVC among patients with ACO, which corroborate previous findings [25]. Interestingly, airway obstruction was less severe in patients with ACO, while previous studies reported decreased [15] or similar [25] FEV_1_ compared to patients with COPD. These discrepancies can be explained by the different patient population in our study: (i) we also included never smokers, (ii) patients were diagnosed by respiratory specialists, and (iii) patients belonged only to GOLD groups C and D. In spite of the higher FEV_1_, dyspnea was as severe in ACO as in COPD (either assessed by mMRC or symptom scores), which might be related to the heightened sensation of dyspnea in this group [26]. We observed a higher prevalence of hypertension and arrhythmia in COPD than in ACO as also shown by others [12].

We did not find a difference in CAT scores between patients with COPD and ACO, which supports previous findings in a cohort with similar symptom burden [27]. Nonetheless, Kurashima et al. found higher CAT scores in Japanese patients with ACO who showed higher scores for cough, phlegm, and dyspnea, but the average CAT score and exacerbation frequency were lower than in our cohort [26]. Furthermore, Park et al. did not show a difference in the disease-specific quality of life between ACO and COPD in a Korean cohort using the CAT tool; however, the Saint George's Respiratory Questionnaire (SGRQ) could detect better HRQL in ACO [12]. On the contrary, ACO was associated with a worse health status assessed by the SGRQ in a US population comprised of patients with different ethnic groups [25]. These and our data suggest that besides exacerbation history, disease-specific health status in ACO might also be influenced by the genetic background of patients.

Our study provides novel data on the relationship between inhaled therapies and HRQL in ACO. We found that patients on dual long-acting bronchodilators have worse CAT scores compared to patients with combination therapies including ICS. This supports the use of ICS in ACO as also stated in the consensus-based document available in the Global Strategy for Asthma Management and Prevention report [10].

Our results demonstrate that different clinical factors modulate disease-specific quality of life in COPD and ACO with exacerbations. The negative influence of smoking history [28], the intensity of dyspnea [29], and the exacerbation number [30] in COPD have already been known, but the unfavorable effect of gastroesophageal reflux disease on CAT score has not been described before. It can be speculated that GERD worsens cough, which is also associated with higher CAT in our cohort; however, further research should give better insight into the mechanism of GERD and HRQL. Contrary to COPD, dyspnea is not associated with CAT scores in ACO, which was rather related to cough as also noted by others [26]. In ACO, the number of severe exacerbations is not associated with higher CAT scores, which could be explained by better recovery induced by the better response to systemic steroids in this population compared to patients with COPD. The association of the frequent use of reliever medications and arrhythmia to CAT scores in ACO might be linked, and it raises attention to the speculative negative effects of reliever abuse on disease-specific HRQL.

The EQ-5D VAS scores were low for both ACO and COPD confirming similarly reduced health status in these groups. This extends previous findings, which showed that patients with ACO have reduced health status, comparable to COPD patients of the exacerbator phenotype [31]. Interestingly, most patients reported some problems in all five dimensions of the EQ-5D, and there was a tendency for a higher proportion of COPD patients with any problems of depression and anxiety. In a Polish COPD cohort, an increased prevalence of both anxiety and depression was noted for frequent exacerbators compared to patients with ACO [32], which is verified in our cohort of exacerbation-matched patients.

The factors influencing generic health, as reflected by EQ-5D VAS scores, in patients with ACO and COPD are partly shared. We show that contrary to smoking status, which does not influence HRQL in this and in other COPD cohorts [7], pack-years do exert a negative influence on EQ-5D VAS score in both groups. Dyspnea at rest is the only symptom related to health status both in COPD and ACO, which might be partly explained by the altered dimensions of dyspnea in patients with COPD [29]. Prebronchodilator FEV_1_ did not show a relation to HRQL; however, other lung function values including specific airway resistance and readouts of hyperinflation could better evaluate the beneficial effect of bronchodilators on dyspnea and quality of life [33], but these parameters were not measured in our study. We corroborated previous findings on the negative effect of cardiovascular comorbidities in COPD [2] by showing that HRQL is associated with cardiovascular comorbidities including arrhythmia (COPD and ACO) and hypertension (ACO). Like others [4], we also found that female patients have lower HRQL if they have COPD, but not ACO. However, higher classes of education are associated with better HRQL in both groups as already shown for COPD [7].

We analyzed the correlation between the disease-specific and generic HRQL measures in ACO for the first time. We found that the CAT and EQ-5D VAS scores were more weakly correlated in ACO than in COPD, which can be explained by the higher influence of nonrespiratory symptoms on the general health status in ACO.

Our study was performed in a large cohort of COPD patients diagnosed by respiratory specialists; however, it has few limitations. First, comorbidities which are known to influence HRQL in COPD and might also affect it in ACO such as psychiatric disorders and peripheral artery disease [7] were not studied. Second, comorbidities were self-reported by patients, which might limit their validity. Third, utility score could not be calculated for EQ-5D-3L. Furthermore, we did not measure bronchial reversibility, although a subgroup of patients with COPD alone show a clinically relevant bronchial reversibility linked with inflammatory processes [34]. Importantly, reversibility was related to dyspnea and quality of life in a recent COPD cohort [35].

## 5. Conclusions

In conclusion, patients with COPD and ACO experiencing exacerbations have similarly reduced disease-specific and generic qualities of life, but patients with COPD seem to have more severe problems in the dimension of anxiety and depression. Pack-years and the frequent use of short-acting bronchodilators exert a negative effect on HRQL measures both in ACO and COPD. HRQL is influenced by symptoms and comorbidities as well. Low HRQL in COPD is associated with the female gender, dyspnea, cough, GERD, and arrhythmia, while it is related to arrhythmia, hypertension, and cough, but less to dyspnea in ACO. Our results aid the development of complex therapeutic strategies to improve the health status of patients with COPD and ACO.

## Figures and Tables

**Table 1:** Demographic and clinical data of patients with COPD and ACO.

	COPD (*n* = 705)	ACO (*n* = 148)	*p* value
Men	50.9%	42.6%	0.065
Age (years)	63.7 ± 9.5	62.3 ± 11.2	0.109
Body mass index (kg/m^2^) (all patients)	27.8 ± 6.6	29.0 ± 6.2	0.043
Body mass index (categories)			0.048
<20	9.9%	5.4%	0.082
20-24.9	28.6%	21.6%	0.087
25.0-29.9	27.9%	30.4%	0.549
>30	33.6%	42.6%	0.038
Smoking status			<0.001
Current	54.5%	39.2%	<0.001
Past	36.0%	35.1%	0.834
Never	9.5%	25.7%	<0.001
Pack-years	32.3 ± 17.5	31.7 ± 19.4	0.717
Educational level			0.104
Low	44.4%	43.9%	
Medium	51.1%	47.3%	
High	4.5%	8.8%	
No. of hospitalizations and ER visits due to respiratory symptoms in the past year			0.932
0	62.4%	60.8%	
1	27.7%	29.1%	
≥2	9.9%	10.1%	
FVC (L)	2.22 ± 0.70	2.30 ± 0.88	0.239
FVC (% reference)	69.4 ± 17.6	71.3 ± 18.3	0.237
FEV_1_ (L)	1.25 ± 0.46	1.34 ± 0.58	0.040
FEV_1_ (% reference)	48.6 ± 14.9	52.3 ± 16.2	0.007
FEV_1_/FVC	0.56 ± 0.10	0.58 ± 0.10	0.037
Inhaled therapy			<0.001
LAMA	3.1%	1.4%	0.238
LABA	0.7%	0%	0.303
LAMA+LABA	49.2%	25.7%	<0.001
LABA+ICS	3.3%	16.9%	<0.001
LABA+LAMA+ICS	43.7%	56.1%	0.006
Comorbidities			
Gastroesophageal reflux	22.4%	21.0%	0.696
Ischemic heart disease	22.4%	18.9%	0.350
Hypertension	60.9%	52.7%	0.066
Myocardial infarction	4.5%	2.7%	0.312
Arrhythmia	10.8%	6.1%	0.083
Heart failure	7.8%	5.4%	0.311
Cerebrovascular event	5.7%	4.7%	0.647
Osteoporosis	7.4%	9.5%	0.646
Diabetes	12.3%	14.2%	0.167
Glaucoma	2.0%	0.7%	0.270
Benign prostate hyperplasia	13.4%	11.1%	0.623
Total number of comorbidities	1.63 ± 1.49	1.40 ± 1.31	0.090

Data are shown as mean ± SD. Data were analyzed with Fisher's exact test, *t*-test, and *χ*^2^ tests (with post hoc analysis). ACO: asthma-COPD overlap; ER: emergency; FVC: forced vital capacity; FEV_1_: forced expiratory volume in the 1^st^ second; LAMA: long-acting muscarinic antagonist; LABA: long-acting beta_2_-agonists; ICS: inhaled corticosteroid.

**Table 2:** Symptoms and quality of life in patients with COPD and ACO.

	COPD	ACO	*p* value
CAT score	20.7 ± 6.7	21.1 ± 6.3	0.522
EQ-5D VAS score	56.2 ± 17.8	53.7 ± 18.2	0.110
EQ-5D scores			
Mobility	1.54 ± 0.52	1.51 ± 0.54	0.511
Self-care	1.48 ± 0.56	1.48 ± 0.58	0.928
Usual activities	1.83 ± 0.55	1.89 ± 0.53	0.304
Pain/discomfort	1.74 ± 0.57	1.69 ± 0.55	0.318
Anxiety/depression	1.76 ± 0.66	1.66 ± 0.66	0.074
mMRC score	2.5 ± 0.8	2.4 ± 0.8	0.692
Severity of symptoms in the past week			
Dyspnea at rest	3.64 ± 3.06	3.67 ± 3.33	0.900
Dyspnea during daily activities	4.27 ± 2.98	4.60 ± 2.92	0.216
Cough	5.09 ± 2.19	4.93 ± 2.41	0.425
Sputum production	1.67 ± 0.64	1.69 ± 0.64	0.738
Use of short-acting bronchodilators in the past week (% all patients)			0.274
≤1	22.5%	20.4%	
2-3	29.5%	23.1%	
>3	48.0%	56.5%	

Data are shown as mean ± SD. Data were analyzed with *t*-test, Mann-Whitney test, and *χ*^2^ test. CAT: COPD Assessment Test; mMRC: Modified British Medical Research Council Questionnaire.

**Table 3:** Quality of life measures according to maintenance inhaled therapy.

	CAT		*p* value	EQ-5D VAS score		*p* value
	COPD	ACO		COPD	ACO	
LAMA+LABA	20.5 ± 6.9	23.0 ± 6.3	0.033	59.4 ± 16.1	59.5 ± 15.3	0.963
LABA+ICS	20.4 ± 6.0	20.4 ± 6.0	0.958	50.9 ± 23.9	47.8 ± 18.4	0.619
LABA+LAMA+ICS	20.9 ± 6.8	20.4 ± 6.3	0.545	52.9 ± 18.3	53.4 ± 18.3	0.853

Data are shown as mean ± SD. Data were analyzed with *t*-test.

**Table 4:** Estimates yielded from multiple regression analysis to predict factors associated with quality of life measures.

	COPD		ACO	
	CAT	EQ-5D VAS	CAT	EQ-5D VAS
Age	-0.018	0.021	0.031	0.171
Gender				
Male	Ref.	Ref.	Ref.	Ref.
Female	0.403	-3.617^a^	0.177	-4.327
Body mass index (kg/m^2^)				
<20	0.549	1.820	-1.351	-0.773
20-24.9	Ref.	Ref.	Ref.	Ref.
25.0-29.9	0.863	-2.160	-1.891	-2.669
>30	0.769	-0.471	-3.407^d^	-7.136
Smoking status				
Current	0.781	0.653	-0.034	-0.491
Ex-smoker	Ref.	Ref.	Ref.	Ref.
Pack-years	0.029^a^	-0.094^a^	-0.006	-0.238^a^
Educational level				
Low	Ref.	Ref.	Ref.	Ref.
Medium	-0.452	4.208^b^	-0.594	3.009
High	-0.903	1.441	-1.046	10.372^e^
No. of hospitalizations and ER visits due to exacerbation in the past year				
0	Ref.	Ref.	Ref.	Ref.
1	-0.722	0.058	-2.598	-0.262
≥ 2	2.257^b^	-0.443	-6.772^a^	-6.430
FEV_1_ (% reference)	-0.024	0.072	-0.049	0.143
Comorbidities				
Absence of comorbidity	Ref.	Ref.	Ref.	Ref.
Gastroesophageal reflux	1.578^b^	1.118	0.477	4.149
Ischemic heart disease	-1.104^d^	0.979	-0.476	-0.198
Hypertension	0.098	2.473	1.249	-6.218^e^
Past myocardial infarction	1.409	5.494	9.143	-9.305
Arrhythmia	0.674	-6.298^a^	5.589^e^	-15.852^a^
Heart failure	0.830	-0.031	0.993	-7.320
Past cerebrovascular event	-0.404	-3.174	-1.922	-6.255
Osteoporosis	-1.508	0.213	-0.183	4.052
Diabetes	-0.431	-0.235	-3.067	-1.123
Glaucoma	2.869	-1.372	-^1^	-^1^
Chronic bronchitis	-1.341^a^	2.583	-^2^	-^2^
Severity of symptoms in the past week				
Dyspnea at rest	0.573^c^	-1.286^c^	0.150	-1.302^d^
Dyspnea during daily activities	0.353^c^	-0.398	0.351	0.808
Cough	0.508^c^	0.405	0.939^a^	-0.143
Sputum production, amount small	Ref.	Ref.	Ref.	Ref.
Medium	0.331	-2.587	0.120	1.664
Significant	0.307	-0.706	-1.806	-8.841
Use of short-acting bronchodilators in the past week (% all patients)				
≤1	Ref.	Ref.	Ref.	Ref.
2-3	0.006	2.258	3.870	-11.269^a^
>3	1.194	-3.831^d^	4.593^a^	-6.393

^a^ *p* < 0.05, ^b^*p* < 0.01, ^c^*p* < 0.001, ^d^*p* = 0.07, ^e^*p* = 0.09. 1: not analyzed due to missing data; 2: not assessed in the ACO group; Ref.: reference category.

## Data Availability

The datasets used and/or analyzed during the current study are available from the corresponding author on reasonable request. Clinical data collection was managed by Adware Research Ltd.
